# Liposomal Resveratrol and/or Carvedilol Attenuate Doxorubicin-Induced Cardiotoxicity by Modulating Inflammation, Oxidative Stress and S100A1 in Rats

**DOI:** 10.3390/antiox9020159

**Published:** 2020-02-16

**Authors:** Abeer M. Alanazi, Laila Fadda, Ahlam Alhusaini, Rehab Ahmad, Iman H. Hasan, Ayman M. Mahmoud

**Affiliations:** 1 Department of Pharmacology and Toxicology, College of Pharmacy, King Saud University, Riyadh 11451, Saudi Arabia; aalanazi22@ksu.edu.sa (A.M.A.); lfadda@ksu.edu.sa (L.F.); reali@ksu.edu.sa (R.A.); ihasan@ksu.edu.sa (I.H.H.); 2 Physiology Division, Zoology Department, Faculty of Science, Beni-Suef University, Beni-Suef 62514, Egypt

**Keywords:** doxorubicin, S100A1, carvedilol, phenols, oxidative stress, cardiomyopathy

## Abstract

Doxorubicin (DOX) is a cytotoxic anthracycline antibiotic and one of the important chemotherapeutic agents for different types of cancers. DOX treatment is associated with adverse effects, particularly cardiac dysfunction. This study examined the cardioprotective effects of carvedilol (CAR) and/or resveratrol (RES) and liposomal RES (LIPO-RES) against DOX-induced cardiomyopathy, pointing to their modulatory effect on oxidative stress, inflammation, S100A1 and sarco/endoplasmic reticulum calcium ATPase2a (SERCA2a). Rats received CAR (30 mg/kg) and/or RES (20 mg/kg) or LIPO-RES (20 mg/kg) for 6 weeks and were challenged with DOX (2 mg/kg) twice per week from week 2 to week 6. DOX-administered rats exhibited a significant increase in serum creatine kinase-MB (CK-MB), troponin-I and lactate dehydrogenase (LDH) along with histological alterations, reflecting cardiac cell injury. Cardiac toll-like receptor 4 (TLR-4), inducible nitric oxide synthase (iNOS), tumor necrosis factor (TNF)-α and interleukin (IL)-6 protein expression were up-regulated, and lipid peroxidation was increased in DOX-administered rats. Treatment with CAR, RES or LIPO-RES as well as their alternative combinations ameliorated all observed biochemical and histological alterations with the most potent effect exerted by CAR/LIPO-RES. All treatments increased cardiac antioxidants, and the expression of S100A1 and SERCA2a. In conclusion, the present study conferred new evidence on the protective effects of CAR and its combination with either RES or LIPO-RES on DOX-induced inflammation, oxidative stress and calcium dysregulation.

## 1. Introduction

Doxorubicin (DOX) is a member of the anthracyclines and one of the most effective anti-cancer agents for solid tumors and leukemia [1]. The main antitumor effects of DOX are attributed to inhibition of topoisomerase II that leads to DNA damage and suppression of protein synthesis [2]. However, its use is restricted by its cardiotoxic adverse effects, including arrhythmia and cardiomyopathy which may consequently lead to congestive heart failure (CHF) [3]. Different mechanisms appear to be responsible for DOX cardiotoxicity. One of these mechanisms is the increased production of free radicals that lead to oxidative stress and lipid peroxidation [1]. Other mechanisms include the increase in intracellular iron and suppression of natural antioxidants, such as reduced glutathione (GSH). Furthermore, DOX can activate inducible nitric oxide synthase (iNOS) and NO production that can provoke cardiomyocyte apoptosis [4]. Moreover, DOX provokes apoptosis either by up-regulating the expression of p53 and caspase-3 [5] or by increasing intracellular calcium level [6]. DOX also stimulates the inflammatory process and increases the levels of tumor necrosis factor-alpha (TNF-α) [7].

Toll-like receptors (TLRs) are important for initiating the innate immune responses [8]. TLRs in the heart are expressed mainly in cardiac myocytes, smooth muscle cells and endothelial cells [9]. TLR-4 is considered to be the main member of the TLRs implicated in DOX-induced cardiomyopathy that initiates left ventricles (LV) damage [7]. Besides TLR-4, TLR-2 has been reported to play a role in regulating inflammation and apoptosis in the heart after DOX administration [10]. Activation of TLR-4 by a ligand stimulates nuclear factor-kappa B (NF-κB) transcriptional activity and the liberation of NF-κB from its inhibitor IkBα and translocation into the nucleus. Activated NF-κB promotes the production of pro-inflammatory cytokines, such as, TNF-α and interleukin (IL)-6 [11,12]. Riad et al have reported that DOX causes oxidative stress that stimulates NF-ĸB, which enhances the inflammatory pathway, and demonstrated the crucial role of the TLR-4/NF-ĸB pathway in LV dysfunction in DOX-induced cardiomyopathy [7].

S100 proteins are Ca^2+^ binding proteins of the EF-hand type that can be expressed in many tissues and play important functions. Various heart diseases are related to S100 proteins which are considered as new markers for cardiac toxicity [13]. S100A1 protein improves cardiac contractility and its expression is reduced following cardiac damage [14]. Human cardiomyopathy exhibited down-regulation of S100A1 expression that decreased the performance of myocardial contraction as documented by Remppis et al [15]. Additionally, Ritterhoff’s group revealed that the mRNA of S100A1 was decreased following dilated and ischemic human cardiomyopathy [16]. Reduced mRNA expression of sarcoplasmic/endoplasmic reticulum calcium ATPase2a (SERCA2a) is a characteristic feature of heart failure (HF). SERCA2a is the main protein involved in calcium reabsorption during relaxation and its low level causes a rise in diastolic calcium [17]. Brinks et al reported that gene therapy with S100A1 in cardiac cell dysfunction enhanced SERCA2a activity 2-fold [18]. During diastole, S100A1 augments SERCA2a function that reduces sarcoplasmic reticulum (SR) Ca^2+^ sparks and raises SR Ca^2+^ load leading to cardiac relaxation; while in systole, S100A1 elevates both SR Ca^2+^ load and ryanodine receptor 2 (RyR2) opening that mediates a high level of intracellular Ca^2+^ transients and supports heart contractile function [16].

Several treatments are used as cardioprotective agents against DOX-induced cardiomyopathy. β-blockers such as carvedilol (CAR) are examples of these drugs [19]. CAR possesses an antioxidant effect and inhibits oxygen radical generation, which is related to its carbazole moiety [20]. Furthermore, CAR restores calcium dysregulation by the enhancement of SERCA2a activity in myocytes [21]. The anti-inflammatory action of CAR was documented in adriamycin-induced cardiotoxicity where CAR significantly attenuated the TNF-α/NF-κB pathway and consequently cyclooxygenase 2 (COX2) and IL-6 expression [22]. However, CAR enhanced hepatic dysfunction and protected against cardiotoxicity induced by DOX as confirmed by the biochemical and histopathological examinations of the hepatic and cardiac tissues [23]. The protective effect of CAR against DOX cardiomyopathies has been reported in rodent models as well as in humans [24,25,26,27]. Given its ability to selectively block β_1_-adrenoceptor (AR), CAR improves heart function and is effective in the treatment of HF [28]. In contrast, the beneficial role of β_2_-AR blockade in patients with HF is debated [28]. Previous reports have demonstrated a moderate β_1_-AR selectivity and a slight β_2_-selectivity of CAR [29]. However, CAR can block β_2_-AR more selectively than β_1_ and accumulate in the cardiac tissue [30]. The higher selectivity towards β_2_-AR resulted in persistent blockade of these receptors and contributed to the beneficial effects of CAR in HF [30]. Therefore, CAR may preferentially inhibit arrhythmias and other harmful effects of adrenaline [30]. Additionally, CAR prevented tissue injury and decreased β_3_-AR expression in the ventricle of diabetic rats subjected to myocardial infarction [31].

Resveratrol (RES) is a small natural polyphenol product with the chemical structure 3,5,4ʹ-trihydroxystilbene [32]. RES plays a protective role against cardiovascular disease (CVD) that is documented by its action against oxidation, inflammation and thrombus aggregation [33]. Vella et al reported that RES has multiple pharmacological actions, including anti-inflammatory and antioxidant activities. Furthermore, it reduces the left ventricular remodeling and dysfunction [34]. The anti-inflammatory action of RES is mediated via inhibition of lipopolysaccharide (LPS)-TLR-4/NF-κB pathway [35,36]. RES affects many calcium signaling pathways in the cardiac cells. These pathways mediate different mechanisms that control calcium influx, release and other calcium sensitive molecules. Previous studies have reported that RES improved heart function and reduced cardiac hypertrophy, which was attributed to silent information regulator 1 (SIRT1) protein that enhanced SERCA2a expression [32,37]. Additionally, RES inhibited l-type Ca^2+^ channels which are the main plasma membrane targets of RES mediating low extracellular Ca^2+^ influx. In normal conditions, Ca^2+^ enters the cell by l-type Ca^2+^ channels which facilitates potassium ion efflux. RES indirectly causes a reduction of potassium efflux and relaxation in the endothelium. The effect of RES on potassium efflux is responsible for its effects on hypertension and arteriosclerosis reduction [37]. Although RES can prevent oxidative stress and inflammation, there is a disagreement regarding its beneficial effects on cardiovascular markers and endothelial dysfunction and in type 2 diabetes patients. The contrasting effects of RES reported in different studies could be explained in terms of different factors, including doses, administration medium and form, age, gender, health status of the intestinal microbiota and its pharmacokinetics [38]. The pharmacokinetic properties of RES are less acceptable and limit its success. RES has poor bioavailability, inadequate water solubility, is chemically unstable and rapidly metabolized in the body with a very short half-life [39]. In order to overcome these pharmacokinetic limitations, a drug delivery system using liposomes becomes an excellent option in order to provide many advantages for the enhancement of RES bioavailability. In this study, we investigated the cardioprotective effect of CAR and/or RES or liposomal (LIPO)-RES against DOX-induced cardiotoxicity with emphasis on inflammation, oxidative stress and calcium dysregulation.

## 2. Materials and Methods

### 2.1. Chemicals

RES and CAR raw powders were purchased from Sigma (St. Louis, MO, USA). DOX (Ebewe Pharma Co, Unterach Am Attersee, Austria) was obtained from a local pharmacy in Riyadh (Saudi Arabia) and marketed liposomal Trans RES^®^ (particle size = 200 nm) was purchased from Lipolife^®^ (Drakes Lane, Chelmsford, UK). Primary antibodies for S100A1 (ab4066), iNOS (ab15323) and GAPDH (ab9483) were purchased from Abcam^®^ (Cambridge, MA, USA). The primary antibody for TLR-4 (NB100-56580) was obtained from Novus Biologicals^®^ (Centennial, CO, USA). The primary antibody for horseradish peroxidase (HRP) conjugated secondary antibody (sc-516102) was obtained from Santa Cruz Biotechnology (Dallas, TX, USA). All other chemicals were of analytical grade and obtained from standard commercial sources.

### 2.2. Animal and Experimental Design

Adult male 8-week old albino Wistar rats weighing 150–180 g were supplied by the Animals Care Centre at the College of Pharmacy, King Saud University (Riyadh, Saudi Arabia). The rats were housed in standard cages and adapted to laboratory conditions (temperature 23 ± 2 °C with 12 h light/dark cycle) for one week prior to the experiment. They were provided food and water ad libitum. The experimental protocol was conducted according to the Research Ethics Committee at King Saud University (Ref. No: KSU-SE-18-31).

Forty-two rats were randomly allocated into seven groups (*n* = 6) as following:

Group I (Control): received 1% carboxy methylcellulose (CMC) as the vehicle of the drugs orally for 6 weeks and intraperitoneal (i.p.) injection of physiological saline twice/week from week 2 to week 6.

Group II (DOX): received 1% CMC orally for 6 weeks and DOX (2 mg/kg i.p.) twice/week for 5 weeks (from week 2 to 6) to produce a total cumulative dose of 20 mg/kg [40].

Group III (CAR): received CAR (30 mg/kg) orally for 6 weeks [41] and DOX as per group II.

Group IV (RES): received RES (20 mg/kg) orally for 6 weeks [42,43] and DOX as per group II.

Group V (CAR/RES): received CAR (30 mg/kg) and RES (20 mg/kg) orally for 6 weeks and DOX as per group II.

Group VI (LIPO-RES): received LIPO-RES (20 mg/kg) orally for 6 weeks and DOX as per group II.

Group VII (CAR/LIPO-RES): received CAR (30 mg/kg) and LIPO-RES (20 mg/kg) orally for 6 weeks and DOX as per group II.

CAR, RES and LIPO-RES were dissolved in 1% CMC. At the end of week 6, all rats were anesthetized, and blood was collected and centrifuged at 3000 rpm for 20 min at 4 °C to separate serum. The rats were dissected, and hearts were removed, washed and parts of the LV were fixed in 10% neutral buffered formalin whereas other parts were frozen in liquid nitrogen and stored at −80 °C.

### 2.3. Assay of Markers of Cardiac Injury, SERCA2a and Cytokines

Serum CK-MB and troponin-I were assayed using ELISA kits (MyBioSource, San Diego, CA, USA) and LDH was determined using a kit supplied by Randox (Crumlin, UK). To assay cardiac SERCA2a, TNF-α and IL-6, samples from the LV were homogenized (10% *w*/*v*) in cold phosphate buffered saline (PBS) with proteinase inhibitors, centrifuged and the supernatant was separated. Protein content in the supernatant was assayed using Bradford reagent [44] and SERCA2a, TNF-α and IL-6 were determined using ELISA kits (MyBioSource, San Diego, CA, USA), following the provided instructions.

### 2.4. Assay of Lipid Peroxidation, GSH and Superoxide Dismutase (SOD)

The levels of malondialdehyde (MDA), a marker of lipid peroxidation, and GSH were measured according to the methods of Ohkawa et al [45] and Beutler et al [46], respectively. SOD activity was assayed following the method of Marklund and Marklund [47].

### 2.5. Histology and Immunohistochemistry

Samples of the LV fixed in 10% neutral buffered formalin were dehydrated and embedded in paraffin wax. The blocks were cut into 5-μm sections which were subjected to deparaffinization, rehydration and staining with hematoxylin and eosin (H&E) for examination using a light microscope. To determine the expression levels of TLR-4 and iNOS, the ABC technique was used as previously described [48]. Briefly, the sections were deparaffinized, rehydrated and washed under tap water and incubated in 3% hydrogen peroxide (H_2_O_2_) for 10 min. The sections were blocked with 5% bovine serum albumin in Tris-buffered saline (TBS) and incubated with anti-TLR-4 or anti-iNOS (1:100 dilution). The sections were washed 3 times in TBS and incubated with secondary antibodies. After washing in TBS, the peroxidase activity was developed using diaminobenzidine followed by hematoxylin counter-staining. The sections were washed in water, dehydrated, cleared, mounted and examined. Quantitative analysis of TLR-4 and iNOS immunostaining was performed using ImageJ (version 1.32j, NIH, USA) and the results are expressed as percent of control.

### 2.6. Western Blotting

To determine the effect of DOX and CAR and/or RES and LIPO-RES on cardiac S100A1 expression, samples from the LV were homogenized in RIPA buffer with proteinase inhibitors, centrifuged and the supernatant was separated. Forty µg total protein was subjected to 12% SDS/PAGE and the separated bands were transferred to a nitrocellulose membrane. After blocking in 5% skimmed milk in TBST, the membranes were probed with anti-S100A1 overnight at 4 °C or anti-GAPDH for 1 h at room temperature. The membranes were washed with TBST and incubated with secondary antibodies and then washed. Thereafter, the membranes were developed using a Bio-Rad ECL kit, scanned, and the band intensity was determined using ImageJ (version 1.32j, NIH, USA).

### 2.7. Statistical Analysis

The data were expressed as mean ± standard error of the mean (SEM). The results were analyzed using one-way ANOVA followed by Tukey’s test on GraphPad 7 (GraphPad Software Inc., La Jolla, CA, USA). A *p* value ≤ 0.05 was considered significant.

## 3. Results

### 3.1. CAR, RES and LIPO-RES Prevent DOX-Induced Cardiac Injury

**Figure 1:**
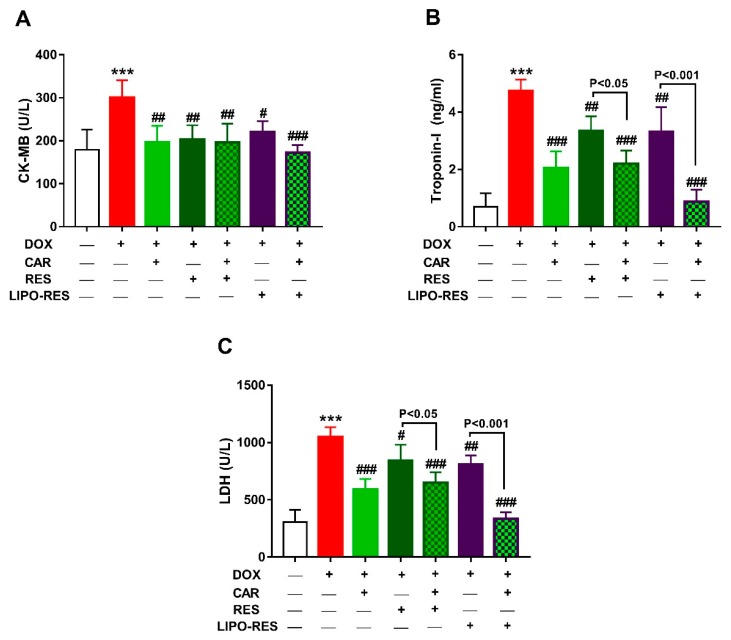
Carvedilol (CAR) and/or resveratrol (RES) and LIPO-RES ameliorate serum CK-MB (**A**), troponin-I (**B**) and LDH (**C**) in DOX-intoxicated rats. Data are expressed as mean ± SEM, (*n* = 6). *** *p* < 0.001 versus Control. ^#^ *p* < 0.05, ^##^ *p* < 0.01 and ^###^ *p* < 0.001 versus DOX.

To evaluate the protective effects of CAR and/or RES and LIPO-RES on DOX cardiotoxicity, we determined serum CK-MB, troponin-I and LDH and conducted a histopathological investigation. DOX caused significant elevation of serum CK-MB (Figure 1A), troponin-I (Figure 1B) and LDH (Figure 1C). Treatment of the DOX-intoxicated rats with CAR, RES or LIPO-RES ameliorated serum troponin-I levels and CK-MB and LDH activities. Treatment with CAR/RES and CAR/LIPO-RES significantly ameliorated troponin-I and LDH when compared with RES and LIPO-RES, respectively.

**Figure 2:**
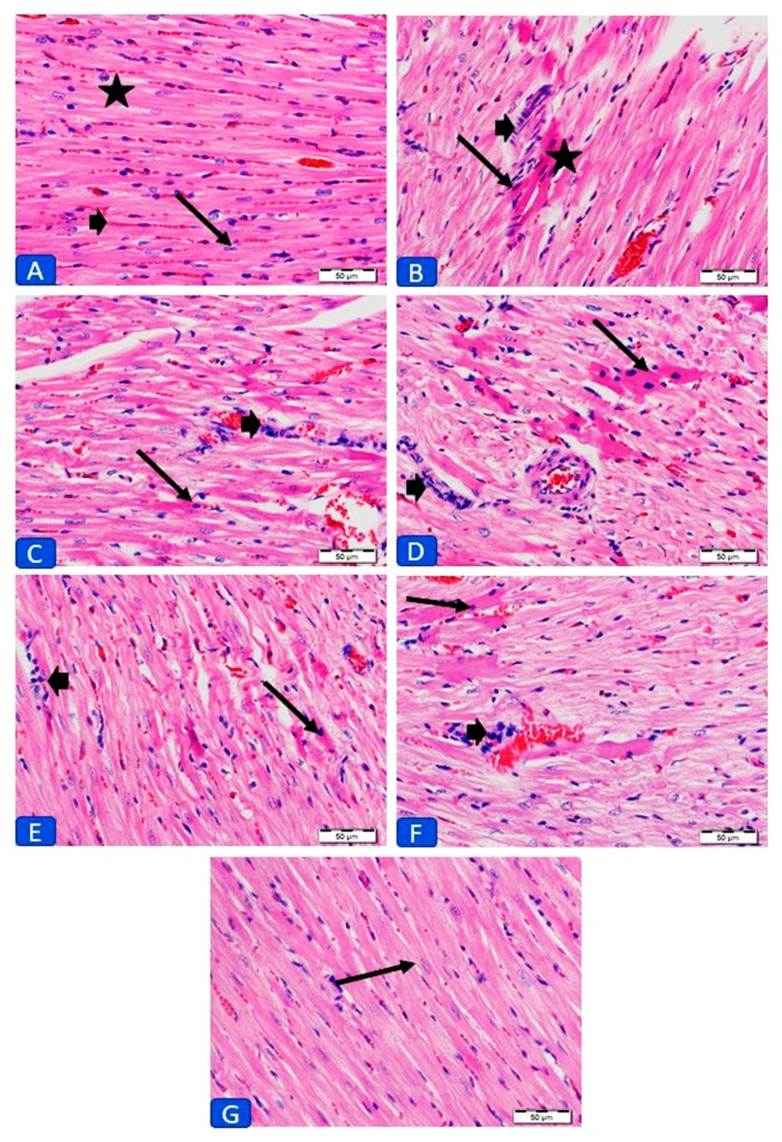
Photomicrographs of sections in the left ventricles (LV) of (**A**) control rats showing normal histology of cardiomyocyte cytoplasm (star) and nuclei (arrows) and normal distribution of endomysium (bold arrow). (**B**) DOX-intoxicated rats showing foci of degenerated cardiomyocytes and collection of inflammatory cells in the endomysium (bold arrow). (**C**) CAR-treated rats showing mild improvement of myocardium with less degeneration (arrow) and inflammatory cells (bold arrow). (**D**) RES-treated rats showing foci of degenerated cardiomyocytes (arrow) and few inflammatory cells (bold arrow). (**E**) CAR/RES- and (**F**) LIPO-RES-treated rats showing moderate decrease in degeneration (arrow) and inflammatory cells (bold arrow); and (**G**) CAR/LIPO-RES-treated rats with no degeneration or inflammatory cells and the cardiomyocytes appear normal. (× 400; H&E; scale bar: 50 µm).

The cardiotoxic effect of DOX was further confirmed by the histopathological examination. While the control rats (Figure 2A) showed normal structure, examination of sections in the heart of DOX-intoxicated rats revealed foci of degenerated myocardium, infiltration of inflammatory cells in the endomysium, along with other manifestations (Figure 2B). Heart sections from DOX-intoxicated rats treated with CAR showed mild cell degeneration (Figure 2C), whereas foci of degenerated myocardium were seen in rats received RES (Figure 2D). Treatment with CAR/RES resulted in moderate improvement with few inflammatory cells (Figure 2E). LIPO-RES supplementation resulted in moderate decrease in degenerated myocardium cells with inflammatory cellular infiltration (Figure 2F), whereas its combination with CAR resulted in the absence of cellular degeneration and inflammatory cells, and the cardiomyocytes appear normal (Figure 2G).

### 3.2. CAR, RES and LIPO-RES Attenuate Cardiac Inflammation in DOX-Intoxicated Rats

Stimulation of TLR-4 has been reported to decrease cardiomyocyte contractility and provoke the activation of NF-κB-dependent inflammatory response, resulting in increased expression of TNF-α, IL-6, iNOS and other inflammatory mediators [11]. Therefore, we determined the effect of CAR and/or RES and LIPO-RES on the expression of TLR-4, TNF-α, IL-6 and iNOS in the heart of DOX-intoxicated rats.

**Figure 3:**
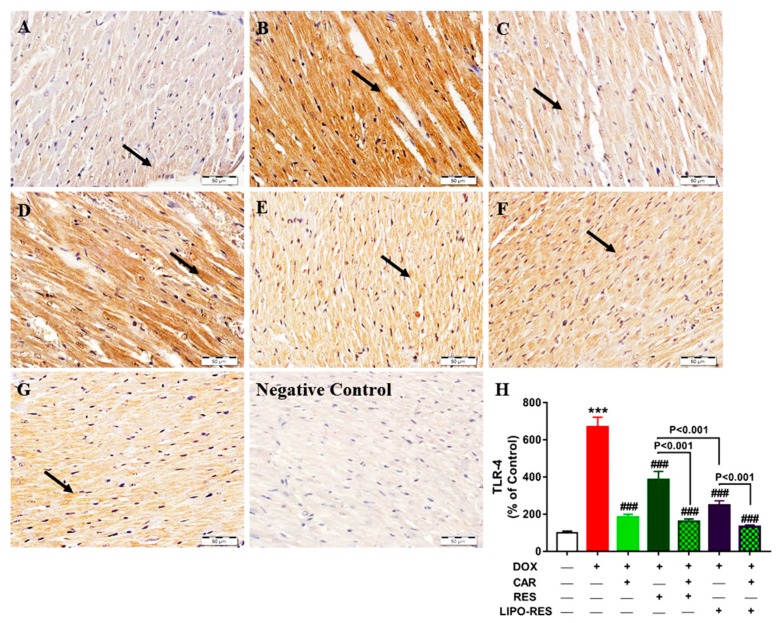
Photomicrographs of anti-TLR-4-stained LV sections in: (**A**) Control rats showing the absence of immune reaction in cardiomyocytes cytoplasm and nuclei (arrow); (**B**) DOX-intoxicated rats showing very strong immune positivity; (**C**) CAR-treated rats showing mild immune positive reaction; (**D**) RES-treated rats showing strong positive immune reaction; (**E**) CAR/RES-treated rats showing very mild immunostaining; (**F**) LIPO-RES-treated rats showing moderate immune reaction and (**G**) CAR/LIPO-RES-treated rats showing marked decrease in TLR-4 immune reactivity. (**H**) Mean ± SEM of TLR-4 immunostaining in LV sections of different groups, (*n* = 6). *** *p* < 0.001 versus Control and ^###^ *p* < 0.001 versus DOX.

Control rats exhibited normal immune staining of TLR-4 in the myocardium as represented in Figure 3A. In contrast, the heart of DOX-intoxicated rats showed very strong immune positivity of TLR-4 in the cardiomyocytes (Figure 3B). Sections in the heart of DOX-intoxicated rats treated with CAR (Figure 3C), RES (Figure 3D) and their combination (Figure 3E) showed mild, strong positive and very mild TLR-4 expression, respectively. Oral supplementation of LIPO-RES (Figure 3F) and its combination with CAR (Figure 3G) resulted in moderate and marked decrease in TLR-4 expression, respectively.

Quantitative analysis of TLR-4 expression revealed a significant up-regulation in DOX-intoxicated rats (*p* < 0.001) when compared with the control group (Figure 3H). CAR, RES and LIPO-RES decreased TLR-4 expression significantly in DOX-intoxicated rats (*p* < 0.001). CAR/RES and CAR-LIPO-RES reduced TLR-4 expression significantly when compared with RES and LIPO-RES, respectively. Additionally, LIPO-RES decreased TLR-4 significantly when compared with RES (Figure 3H).

**Figure 4:**
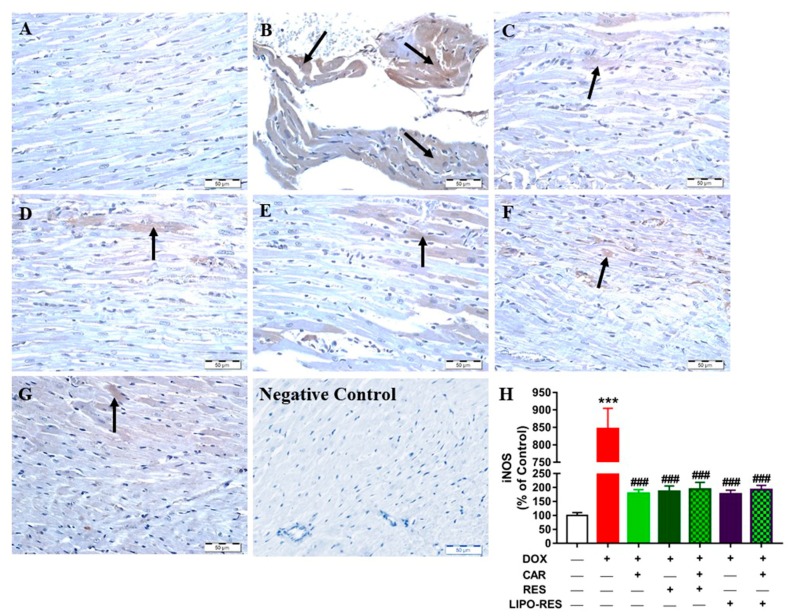
Photomicrographs of anti-iNOS-stained LV sections in: (**A**) Control rats showing the absence of immune reaction in cardiomyocytes cytoplasm and nuclei (arrow); (**B**) DOX-intoxicated rats showing multiple scattered foci of strong immune positivity; (**C**) CAR- (**D**) RES- (**E**) CAR/RES- and (**F**) LIPO-RES-treated rats showing moderate decrease in iNOS expression, and; (**G**) CAR/LIPO-RES-treated rats showing marked decrease in iNOS immune reactivity. (**H**) Mean ± SEM of iNOS immunostaining in LV sections of different groups, (*n* = 6). *** *p* < 0.001 versus Control and ^###^ *p* < 0.001 versus DOX.

Control rats exhibited normal expression of iNOS (Figure 4A) whereas DOX increased its expression markedly (Figure 4B). Sections in the heart of rats received CAR (Figure 4C), RES (Figure 4D), CAR/RES (E), LIPO-RES (Figure 4F) and CAR/LIPO-RES (Figure 4G) showed significant decrease in iNOS expression (Figure 4H). The statistical analysis showed non-significant differences between the different treatments on iNOS expression in the heart of DOX-intoxicated rats (Figure 4H).

**Figure 5:**
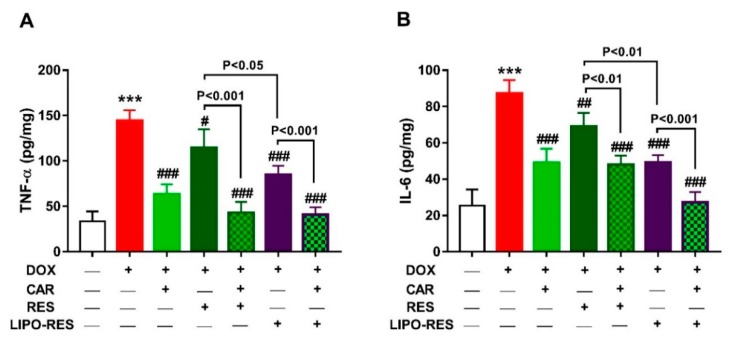
CAR and/or RES and LIPO-RES decrease cardiac TNF-α (**A**) and IL-6 (**B**) in DOX-intoxicated rats. Data are expressed as mean ± SEM, (*n* = 6). *** *p* < 0.001 versus Control. ^#^ *p* < 0.05, ^##^ *p* < 0.01 and ^###^ *p* < 0.001 versus DOX.

The pro-inflammatory cytokines TNF-α (Figure 5A) and IL-6 (Figure 5B) were significantly increased in the heart of rats that received DOX. CAR, RES and LIPO-RES decreased TNF-α and IL-6 significantly in DOX-intoxicated rats. LIPO-RES was more effective in reducing TNF-α (*p* < 0.05) and IL-6 (*p* < 0.01) when compared with RES. The combination of CAR with RES or LIPO-RES reduced TNF-α and IL-6 significantly when compared with the individual drugs.

### 3.3. CAR, RES and LIPO-RES Prevent Oxidative Stress in the Heart of DOX-Intoxicated Rats

**Figure 6:**
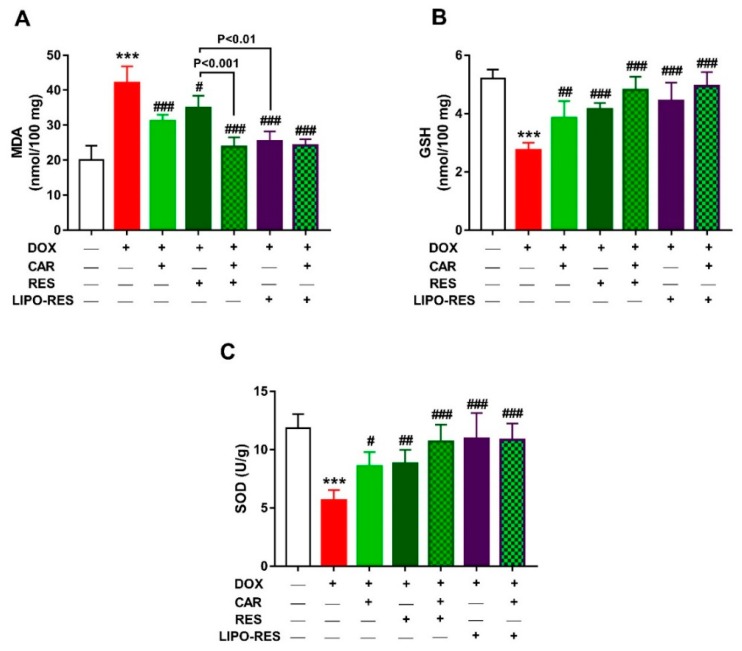
CAR and/or RES and LIPO-RES decrease cardiac MDA (**A**) and increase GSH (**B**) and SOD (**C**) in DOX-intoxicated rats. Data are expressed as mean ± SEM, (*n* = 6). *** *p* < 0.001 versus Control. ^#^ *p* < 0.05, ^##^ *p* < 0.01 and ^###^ *p* < 0.001 versus DOX.

MDA, GSH and SOD were measured to assess the protective effect of CAR, RES and LIPO-RES on DOX-induced oxidative stress. DOX-intoxicated rats exhibited a significant increase in cardiac MDA (Figure 6A) and decreased GSH (Figure 6B) and SOD (Figure 6C). Treatment with CAR, RES and LIPO-RES markedly decreased MDA and increased antioxidants. When compared with RES, LIPO-RES and CAR/RES decreased cardiac MDA levels significantly.

### 3.4. CAR, RES and LIPO-RES Upregulate Cardiac S100A1 and SERCA2a in DOX-Intoxicated Rats

**Figure 7:**
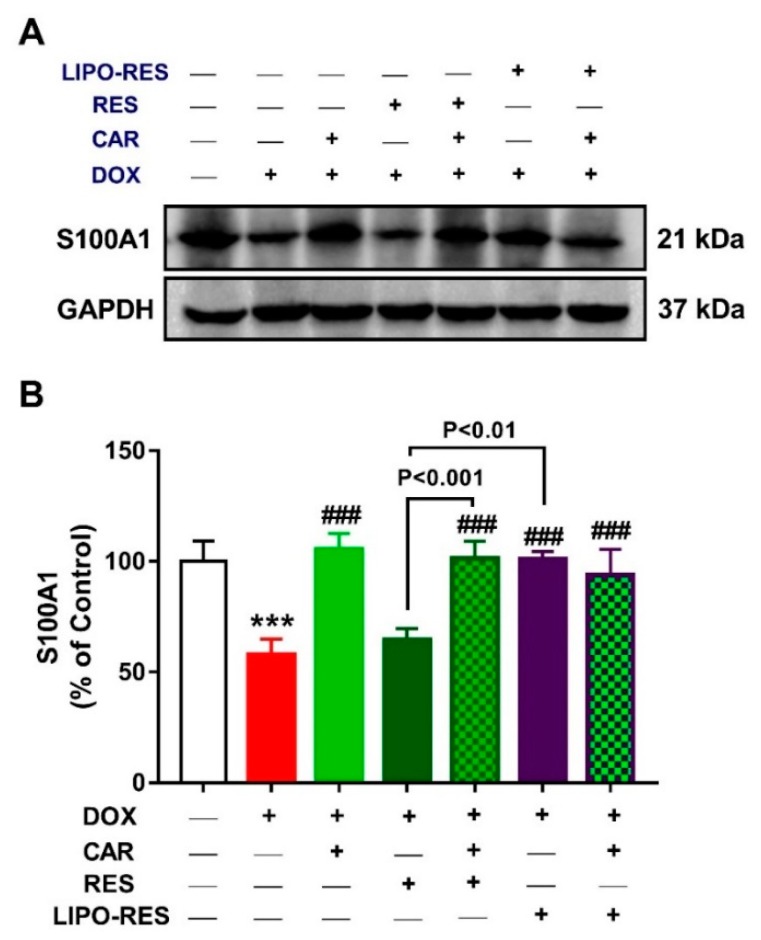
Effect of CAR and/or RES and LIPO-RES on cardiac S100A1. (**A**) Representative Western blotting of S100A1 and GAPDH. (**B**) CAR and/or RES and LIPO-RES up-regulated cardiac S100A1 in DOX-intoxicated rats. Data are expressed as mean ± SEM, (*n* = 6). *** *p* < 0.001 versus Control. ^###^ *p* < 0.001 versus DOX.

**Figure 8:**
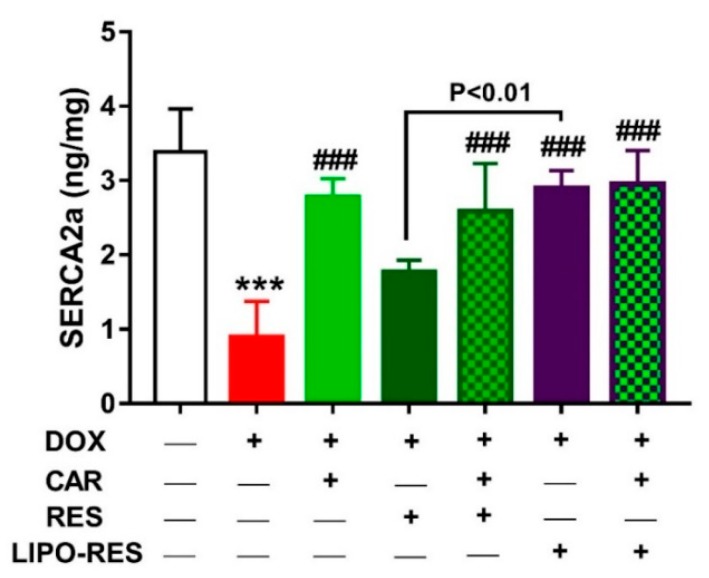
CAR and/or RES and LIPO-RES increase cardiac SERCA2a in DOX-intoxicated rats. Data are expressed as mean ± SEM, (*n* = 6). *** *p* < 0.001 versus Control. ^###^ *p* < 0.001 versus DOX.

Given the role of the Ca^2+^ binding protein S100A1 in improving Ca^2+^ handling and contractile performance of the cardiomyocytes via its interaction with SERCA2a RyR2 [49], we investigated the impact of DOX and the effects of CAR and/or RES and LIPO-RES on the expression levels of S100A1 (Figure 7) and SERCA2a (Figure 8). DOX suppressed S100A and SERCA2a in the heart of rats when compared with the control group (*p* < 0.001). Treatment with RES didn’t improve the expression of both S100A1 and SERCA2a, whereas CAR and LIPO-RES exerted a significant ameliorative effect. In addition, the combination of CAR with RES and LIPO-RES significantly improved the expression of S100A1 and SERCA2a.

## 4. Discussion

DOX is a broad-spectrum antibiotic and antineoplastic agent for hematological and solid tumors. The use of DOX has been limited by the occurrence of dose-dependent toxicity to vital organs, particularly the heart [3]. Although the mechanisms of DOX-induced cardiotoxicity are not fully understood, oxidative stress, intracellular calcium dysregulation, inflammation and apoptosis of cardiomyocytes are the most proposed mechanisms [1]. The challenge for the management of its cardiotoxicity is selecting an agent that has cardioprotective effects and at the same time does not affect its antitumor activity. In this context, CAR showed some promising effects against DOX-induced cardiotoxicity [22]. RES is a polyphenol product possessing important pharmacological effects such as antioxidant- and anti-inflammatory activities [43,50]. It produced cardio-protective effects in different cardiac disorders including DOX-induced cardiomyopathy [19]. However, these treatments do not produce sufficient therapeutic effects. Therefore, there is an urgent need for novel strategies for the control DOX’s cardiac side effects. LIPO-RES is considered as one of these promising cardioprotective approaches [51]. Therefore, we aimed to explore the cardioprotective effects of RES and LIPO-RES alone and in combination with CAR in DOX-intoxicated rats, pointing to their ability to modulate oxidative stress, inflammatory response, S100A1 and SERCA2a.

Herein, the cardiotoxicity of DOX in rats was manifested by elevated serum levels of troponin-I, LDH and CK-MB, and was also confirmed by the histopathological examination, which revealed massive changes in the cardiac tissue, fragmentation and degeneration of cytoplasm and nuclei. These data were supported by previous studies where similar findings have been reported [40,52]. CAR, RES and LIPO-RES reduced the cardiotoxicity induced by DOX administration as evidenced by significant reduction of CK-MB, LDH and troponin-I levels and decreased myocardium degeneration in the histopathological findings that were in line with previous studies [40,53,54]. The combination of CAR and LIPO-RES showed significant reduction in serum LDH and troponin-I and the better enhancement in cardiac architecture that indicated improvement of cardiac injury following DOX administration versus other treated groups. However, RES treatment showed weak ameliorative effects on CK-MB, LDH and troponin-I in the DOX-administered group and this was also confirmed by the histopathological examination which indicated more degeneration of cardiac muscle versus the other treated groups.

Inflammation is one of the critical characteristics of DOX cardiotoxicity. DOX stimulates TLR-4 that consequently activates NF-κB which is necessary for cardiac apoptosis [55,56]. NF-κB is a sensitive transcription factor that controls inflammatory signaling cascades that mediate transcription of important inflammatory mediators, such as COX-2, IL6, iNOS and TNF-α [57,58]. There is a growing evidence revealing that cardiac NO level is raised in cardiotoxicity induced by DOX treatment [59]. The increase in cardiac NO level by DOX is attributed to the overexpression of iNOS that is generated through inflammation. The high levels of NO produce peroxynitrite by reacting with the free radical superoxide anion, then peroxynitrite induces cardiac oxidative damage, apoptosis and lipid peroxidation [4]. In accordance to the previously mentioned data, the present study revealed significant elevation of cardiac TNF-α, IL6 levels, TLR-4 and iNOS protein expression in the DOX-administered group. CAR, RES and LIPO-RES significantly attenuated the myocardial TNF-α level and decreased TLR-4 and iNOS protein expression. These results were in parallel with the findings of previous studies that proved the anti-inflammatory effect of CAR and RES [22,60,61]. Clearly, the combination of CAR and LIPO-RES exerted a more potent anti-inflammatory effect than each individual agent, and the combination CAR/RES showed a better effect than either drug alone on TNF-α levels and TLR-4 protein expression. Therefore, it is noteworthy assuming that CAR has synergistic effects with RES as well as LIPO-RES on TNF-α and TLR-4. Interestingly, concurrent administration of CAR and LIPO-RES exerted a superior effect matched with the other treated groups regarding to TLR-4 pathway that consequently produced low levels of the main cytokines IL-6 and TNF-α which reflect the additive anti-inflammatory effect of this combination. The present results were in line with Mahmoud et al who found that CAR administration significantly decreased iNOS that is mediated via its inhibitory effect on NF-κB expression [62]. One of the possible mechanisms of the cardioprotective effects of RES is the anti-inflammatory action that mediated by its ability to increase the expression of SIRT1 and AMPK proteins [61]. RES inhibits NF-κB inflammatory pathway through stimulating the AMPKa/SIRT1 pathway, thereby alleviating the inflammatory process [63]. SIRT1 controls the transcription of NF-κB and p53. SIRT1 inactivates NF-κB p65 subunit and causes deacetylation of NF-κB that consequently inhibits the transcription of inflammatory cytokines, including IL-6, iNOS and TNF-α [64,65]. Csiszar et al reported that TNF-α induces NF-κB activation, and RES directly attenuates the expression of TNF-α in human coronary arterial cells [66]. The current study showed that the liposomal product enhanced the anti-inflammatory action of native RES as evidenced by the significantly decreased TNF-α and IL-6 in rats received LIPO-RES.

Besides inflammation, oxidative stress has been implicated in cardiotoxicity induced by DOX [40,41]. Here, DOX administration induced oxidative stress evidenced by the significant increase in cardiac MDA levels along with abolished GSH and SOD. Accordingly, previous studies have demonstrated that DOX increases ROS generation mediated via NADPH oxidase and MDA and decreases SOD and catalase activities in the heart of rodents [40]. Excess ROS are well-acknowledged to damage cellular macromolecules, such as proteins, lipids and DNA. In this context, Tatlidede et al have shown increased DNA damage in DOX-intoxicated rats [40]. Therefore, attenuation of oxidative stress plays a role, at least in part, in the protective effects of CAR and/or RES and LIPO-RES. This notion is supported by previous studies showing the ability of CAR and RES to suppress ROS and MDA, and boost antioxidant enzymes in DOX-intoxicated rats [40,41]. Interestingly, LIPO-RES reduced MDA significantly in the heart of DOX-intoxicated rats when compared with RES. These findings support the superior cardioprotective effect of LIPO-RES.

Disturbance of calcium homeostasis is another mechanism involved in DOX-induced cardiac toxicity. Doxorubicinol, a major metabolite of DOX, alters the ability of SR to sequester calcium by interfering with SERCA2 function and stimulates the release of calcium from SR [19]. In the present work, DOX exhibited a significant reduction of SERCA2a that is similar to the results of Zhang et al, who reported that DOX decreased SERCA2a expression in rats [67]. In contrast, concomitant administration of CAR, RES and LIPO-RES significantly restored SERCA2a activity. Of note, LIPO-RES significantly restored SERCA2a when compared with RES. The present results were supported by the finding of Kalay et al, who documented that CAR enhanced SERCA2a activity and increased its gene expression [21]. The effect of CAR on SERCA2a gene transcription is mediated mainly by its effect on specificity protein 1 (Sp1) sites in the SERCA2a gene promoter region [68]. Moreover, the present study is in agreement with previous studies proving the beneficial effect of RES on SERCA2a [69,70]. RES affects SERCA2a gene expression mostly through the SIRT1 transcriptional pathway. SIRT1 function is involved in cardiac contractility via regulating SERCA2a promoter activity [71]. To further explore the ameliorative mechanism of CAR and/or RES and LIPO-RES on DOX cardiotoxicity, the expression of S100A1 was determined. This study is considered the first one that discussed the effect of DOX, CAR, RES and LIPO-RES individually as well as the alternative combinations on S100A1 protein expression. S100A1 exhibits its actions mainly in the heart but is also expressed in skeletal muscle, brain and kidneys [72]. It interacts with both SERCA2a and RyR2, which are important for Ca^2+^ handling and cardiac contraction. S100A1 enhances Ca^2+^ transient and reduces diastolic Ca^2+^ overload by decreasing SR Ca^2+^ leakage to modulate cardiac function [49]. The data of this study revealed that DOX caused down-regulation of S100A1 and that may be considered as one of the causes for the reduction of SERCA2a. Up till now, there has not been enough data to demonstrate the regulation of S100A1 protein expression. Kiewitz et al reported that SP1 elements are involved in the S100A1 promoter region [73]. Additionally, cyclic adenosine monophosphate (cAMP) responsive elements were also detected in the S100A1 gene [73,74]. Previous data showed that Sp1 was down-regulated after DOX administration, and Fatemi et al found that cAMP-stimulating agents ameliorated DOX-induced apoptosis [75]. As a result, DOX may affect S100A1 via decreasing SP1 protein level. In the present work, CAR and LIPO-RES increased S100A1 protein expression while RES showed non-significant increase versus the DOX-administered group. It was expected that CAR may affect S100A1 protein expression as mentioned previously by its effects on SP1 similar pathway to SERCA2 protein. Whereas the beneficial effect of LIPO-RES on S100A1 is suggested to be attributed to its effect on cAMP activation [76]. The beneficial effect of CAR or LIPO-RES on S100A1 may be the reason for the improvement of cardiac contractility and enhancement of the SERCA2 level.

## 5. Conclusions

This study is the first one that evaluated the protective effect of the combination of CAR with either RES or LIPO-RES against inflammation, oxidative stress and calcium dysregulation induced by DOX administration, as well as their effect on S100A1 protein. The present results proved that treatment of DOX-administered rats with either CAR or LIPO-RES alone or together alleviated inflammation, oxidative stress and tissue injury evidenced by biochemical and molecular as well as histopathological studies. The combination of CAR and LIPO-RES clearly exerted the best beneficial effects according to most of the previous aforementioned measured parameters. Concomitant administration of CAR in combination with LIPO-RES could be considered as a promising candidate for protection against DOX cardiotoxicity.
